# An Effective BERT-Based Pipeline for Twitter Sentiment Analysis: A Case Study in Italian

**DOI:** 10.3390/s21010133

**Published:** 2020-12-28

**Authors:** Marco Pota, Mirko Ventura, Rosario Catelli, Massimo Esposito

**Affiliations:** 1 Institute for High Performance Computing and Networking (ICAR), National Research Council, 80131 Naples, Italy; marco.pota@icar.cnr.it (M.P.); mirko.ventura@icar.cnr.it (M.V.); massimo.esposito@icar.cnr.it (M.E.); 2 Department of Electrical Engineering and Information Technologies (DIETI), University of Naples Federico II, 80125 Naples, Italy

**Keywords:** sentiment analysis, NLP, language models, BERT, Italian language

## Abstract

Over the last decade industrial and academic communities have increased their focus on sentiment analysis techniques, especially applied to tweets. State-of-the-art results have been recently achieved using language models trained from scratch on corpora made up exclusively of tweets, in order to better handle the Twitter jargon. This work aims to introduce a different approach for Twitter sentiment analysis based on two steps. Firstly, the tweet jargon, including emojis and emoticons, is transformed into plain text, exploiting procedures that are language-independent or easily applicable to different languages. Secondly, the resulting tweets are classified using the language model BERT, but pre-trained on plain text, instead of tweets, for two reasons: (1) pre-trained models on plain text are easily available in many languages, avoiding resource- and time-consuming model training directly on tweets from scratch; (2) available plain text corpora are larger than tweet-only ones, therefore allowing better performance. A case study describing the application of the approach to Italian is presented, with a comparison with other Italian existing solutions. The results obtained show the effectiveness of the approach and indicate that, thanks to its general basis from a methodological perspective, it can also be promising for other languages.

## 1. Introduction

Blogs, micro-blogs, social networks and all these types of websites are a massive source of information for many analysts, entrepreneurs and politicians who aim to expand their business by exploiting the large amount of text generated by users who give constant and continuous feedback on the visibility of a given subject through feelings, opinions and reviews [1]. In the tourism sector, for example, operators can find solutions to attract new customers and improve the service offered through the analysis of comments and reviews on places of interest [2]. For these reasons, an extensive branch of research aims to develop automatic text classification systems (e.g., aspect extraction [3], opinion mining [4], sentiment analysis [5]), in order to use these data in the best possible way.

In recent years, significant results have been obtained by developing several methods, starting from those based on the creation of rules [6], through those based on machine learning [7], and finally to those based on deep learning [8], which currently represent the state-of-the-art. With particular regard to the field of Natural Language Processing (NLP), language models such as Bidirectional Encoder Representations from Transformers (BERT) [9] achieve outstanding results for text recognition and classification [10], encoding information from text sequences, using a model that has been pre-trained on a huge amount of unlabeled data and fine-tuned on small supervised datasets specifically designed for certain tasks.

Despite the excellent results achieved, the performance of current systems is strictly correlated to the specific language considered, on the one hand, since the available supervised datasets can have different dimensions and include a varying number of elements. On the other hand, the nature of user-generated content on social networks requires further refinements before being processed by these systems.

In the field of sentiment analysis of tweets, most of the scientific literature has obtained state-of-the-art results adopting the approach of training language models directly from scratch starting from corpora made up exclusively of tweets, so that the models could better handle the specific tweet jargon, characterized by a particular syntax and grammar not containing punctuation, with contracted or elongated words, keywords, hashtags, emoticons, emojis and so on. These approaches, working not only in English [11,12], but also in other languages such as Italian [13], Spanish [14,15], and Latvian [16], necessarily impose two constraints: the first requires the building of large corpora of tweets to be used for training the language models in the specific language considered, and the second is the need for substantial resources, of both hardware and time, to train the models from scratch starting from these corpora.

The approach outlined in this article suggests a diverse perspective to mitigate the above constraints, with the following main contributions:

1. A pre-processing phase is carried out to transform Twitter jargon, including emojis and emoticons, into plain text, using language-independent conversion techniques that are general and applicable also to different languages.
2. A language model is used, namely BERT, but in its version pre-trained on plain text instead of tweets. There are two reasons for this choice: firstly, the pre-trained models are widely available in many languages, avoiding the time-consuming and resource-intensive model training directly on tweets from scratch, allowing to focus only on their fine-tuning; secondly, available plain text corpora are larger than tweet-only ones, allowing for better performance.

A case study describing the application of this approach to the Italian language is presented in this paper. The SENTIment POLarity Classification 2016 (SENTIPOLC 2016) [17] Italian dataset has been used, since it has already been experimented with in the most recent state-of-the-art literature [13], offering the possibility of comparison with other Italian methods and systems. In particular, the approach has been instantiated for this case study by fine-tuning and testing the pre-trained language model BERT on the introduced dataset, in its Italian version made available by the MDZ Digital Library team (dbmdz) at the Bavarian State (https://github.com/dbmdz/berts#italian-bert). Even if the approach has been evaluated for the Italian language, it is based on pre-trained language models, which exist for many languages besides Italian, and pre-processing procedures that are essentially language-independent. Given these premises, it has a general basis from a methodological perspective and can be proficiently applied also to other languages.

The paper is structured as follows. Section 2 describes the background and related works. In Section 3, the proposed methodological approach is detailed, while Section 4 describes the experimental design. Results are presented and discussed in Section 5 and Section 6 concludes the work

## 2. Background and Related Works

The purpose of sentiment analysis, also called opinion mining, is to identify people’s attitudes, whether positive, neutral or negative, based on some statement or text they have produced, such as a tweet. In the following, various techniques of artificial intelligence used at the state-of-the-art are reported with particular reference to the analysis of the sentiment in the tweets present on the Twitter platform and appropriately reorganized in datasets.

In detail, in Section 2.1 the models and methods developed over time and used in this research field are detailed, while in Section 2.2 a deepening on pre-processing techniques, often underestimated although very important for the optimization of systems, is proposed. Finally, in Section 2.3, the state-of-the-art in Italian with the most recent advances is detailed.

### 2.1. Techniques for Sentiment Analysis

The reason for the great attention paid in recent years to the field of sentiment analysis by both the industrial and academic community is to be found in the desire to help decision-making processes as much as possible [18]. Consequently, it has been possible to identify two macro-actions to be undertaken. The first one consists in distinguishing objective statements with neutral polarity from subjective ones, and the second one in evaluating the polarity of subjective statements, whether positive or negative; unfortunately, the first action is often ignored, which affects the whole process. However, overall, a large number of approaches proposed by the scientific community for sentiment analysis revolves around these two macro-actions.

The first and most common approaches employed by sentiment analysis are based on features like unigrams, in terms of their presence or frequency, Part Of Speech (POS) tags and term position [19], opinion words and sentences [20], negations [21] and syntactic dependencies [22]. Some approaches have shown effective performance in text categorization, such as Support Vector Machine (SVM) [23], Multinomial Naïve Bayes (MNB) [24] and Maximum Entropy (ME) classifiers and derived ensembles [22,25], even if their classification skills remain limited by the high training costs due to the need for a broader vocabulary, i.e., more words from which more features can be extracted [26] to be used in conjunction with machine learning algorithms for sentiment classification. Finally, one of the most popular multilingual approaches based on machine learning is the SentiStrength tool [27], which, however, does not benefit from special pre-processing techniques for the specific jargon of social networks such as Twitter.

Luckily, in recent years, the field of NLP has begun to develop faster and faster and has become increasingly successful thanks to the combination of two main techniques: word embeddings and deep learning based models, explained in the following. More insight is provided by Zhang et al. [28].

#### 2.1.1. Word Embedding

Word Embedding (WE) is a technique that maps textual tokens, e.g., words, into dense and low-dimensional vector representations, learned on large unlabelled corpora, where each token is related to other tokens in its context.

Word2Vec, proposed by Mikolov et al. [29], was the first technique used for word embedding. Its working mechanism could encode the text through two main approaches: the skip-gram model or the Common Bag Of Words (CBOW) model. While the latter predicts a word on the basis of the words within the surrounding context, the former predicts the words within the surrounding context starting from the current word. These mechanisms map words into vectors that are closer when words are similar and often close together. The Word2Vec approach gained a strong resonance in the scientific world, so much so that it is still used in several areas in conjunction with deep neural networks, such as health care [30]. In the same way Global Vectors (GloVe), proposed by Pennington et al. [31], generate the vector encoding of a word more quickly than Word2Vec because the implementation can be parallelized when launched on a greater amount of data. Moreover, Cao and Rei [32] proposed a novel approach named char2vec based on the representation of characters instead of words.

In the field of sentiment analysis, several specific WE were proposed, based on the prior knowledge acquired through both opinionated words from sentiment lexicons and available sentiment labels. Li et al. [33] proposed a new method for learning word embedding for sentiment analysis based on prior knowledge, which improved the results in comparison with standard WE. Furthermore, Yu et al. [34] presented a new way to refine word embeddings for sentiment analysis using intensity scores from sentiment lexicons. Moreover, Hao et al. [35] applied a novel stochastic embedding technique for cross-domain sentiment classification, preserving the similarity in the embedding space. Finally, Ali et al. [36] proposed a system that retrieved transport content from social networks, representing the documents with word embedding techniques and achieving an effective approach to sentiment classification with 93% accuracy.

#### 2.1.2. Deep Neural Networks

Deep Neural Networks (DNNs) are Artificial Neural Networks (ANNs) that present multiple hidden layers between input and output and exist in a plethora of different architectures depending on the topology of neurons and their connections; among them, some have brought remarkable results over the years in the considered field of research [37]: Convolutional Neural Networks (CNNs), Recurrent Neural Networks (RNNs), up to language models based on transformers [9,38].

On the one hand, CNNs have shown the finest results in computer vision and image processing, and the same architecture has been widely applied to text processing [37]. In this architecture the numerical input is constituted by the pixel value of the image that passes from the input layer to the convolutional layer where filters recognize the researched patterns according to their kernel. Hence the best patterns are chosen by the pooling layer, which associates them with the output. Here a single token can be viewed as a vector by using word embedding, hence a 2D matrix represents the generic sentence. The most famous CNN-based sentiment analysis model was introduced by [39], extensively used by [40] and enhanced by [41]. Furthermore, Chen et al. [8] improved sentiment detection through a two-steps architecture, leveraging separated CNNs trained on sentences clustered according to the number of opinion targets contained. Finally, based on sub-word level information [42], variants for tweet sentiment analysis were tested [43]

On the other hand, RNNs are used for modeling sequential data in a variety of applications. In this architecture, each token in a sentence is processed recurrently and a related hidden state is saved based on all of the previous inputs. Methods based on RNNs fed the sentiment classifier with the complete sentence representation building it with a bottom-up approach [44]. Moreover, the Long Short-Term Memory (LSTM) variant of RNNs [45] is able to handle the vanishing gradient problem of standard RNNs, catching long-term dependencies. Therefore, LSTM networks were proven to perform better that standard RNNs for sentiment analysis [46]. LSTM architecture has obtained the best results in sentiment analysis until 2017 [47]. Alayba et al. [48] have shown the benefits of integrating CNNs and LSTMs, reporting a better accuracy on diverse datasets for Arabic sentiment analysis.

More recently, language models, which consist of a large network previously trained on a large amount of unlabeled data and fine-tuned on down-stream tasks, have made a breakthrough in several natural language understanding tasks.

Howard and Ruder [49] proposed Universal Language Model Fine-Tuning (ULMFiT) and achieved state-of-the-art results in the text classification task. In particular, notable success was gained by transferring the encoder part of an encoder–decoder architecture based on transformers, for constructing task-specific models, such as OpenAI Generative Pre-trained Transformer (GPT) [50] and BERT [9], which is nowadays one of the most popular and best performing models, is available in many languages and investigated with respect to commonalities and differences between language-specific and multilingual versions [51]. In detail, many of the systems proposed to date for sentiment analysis use BERT and its variants, obtaining excellent results. For instance, Sun et al. [10] conducted exhaustive experiments to investigate different fine-tuning methods of BERT on text classification, achieving state-of-the-art results on review sentiment analysis. Moreover, Song et al. [52] explored the potential of BERT intermediate layers to enhance BERT fine-tuning and achieved a new state-of-the-art for aspect-based sentiment analysis.

The most recent works proposed language models specifically pre-trained on tweet corpora: Thakkar and Pinnis [16] achieved encouraging performance leveraging a time-balanced evaluation set for sentiment analysis on Latvian tweets, comparing several BERT-based architectures, and Nguyen et al. [12] presented BERTweet, the first public large-scale pre-trained language model for English tweets; Ángel González et al. [15] proposed TWiLBERT, a specialization of the BERT architecture both for the Spanish language and the Twitter domain. For languages other than English, such as Persian [53] and Arabic [54], recent studies have also focused on deep neural networks such as CNN and LSTM.

### 2.2. Pre-Processing Techniques for Sentiment Analysis

In the field of sentiment analysis, pre-processing techniques are used to rework the text so that it can be better understood by classification systems, for example by reducing noise and reorganizing the content. As a result, along with the development of methods and networks, over the years several pre-processing mechanisms have been progressively implemented and tested by researchers.

One of the first works that dealt with the pre-processing phase was that of Boiy et al. [55], who decided to ignore the pos tagging in the classification phase because of the negative effects it produced on accuracy.

Afterwards, Danisman and Alpkocak [56] thought about a pre-processing approach focused on several aspects: (1) preserving emotion words and negative verbs during the stopword removal phase, (2) replacing short forms with long forms, (3) chaining negative words to emotion words by forming new words, e.g., *not happy* became *NOThappy*, and (4) substituting punctuation with new descriptive words. This approach, as further tested by Agrawal and An [57], also showed that, despite the removal of emotional meaning from some words, stemming improved the accuracy of the classification.

The method proposed by Han and Baldwin [58] to identify and normalize malformed words used a classifier to identify ill-formed words and generated correction candidates based on morpho-phonemic similarity.

In addition, Saif et al. [59] have deepened the use of pre-compiled stop-lists in the field of sentiment analysis on tweets and demonstrated how their use has a negative impact on performance. In the same vein, Angiani et al. [60] have investigated the use of different pre-processing methods showing how stemming is the most effective for sentiment analysis. Looking deeper, Zhao and Gui [61] have distinguished even better the effects of the different pre-processing methods on the accuracy of the classification: while the removal of numbers, stop-words and URLs reduces noise without affecting performance, the replacement of negation and the expansion of acronyms increases performance. Similarly, negations were shown to have more pronounced effects than intensifiers and diminishers, which conversly have almost no effects [62].

Recently, several results have clarified the influence of some techniques on the classification results: in contradiction with Boiy et al. [55], Gratian and Haid [63] showed the usefulness of pos tagging used along with more modern techniques, and Pecar et al. [64] underlined the effectiveness of emoticons’ pre-processing in combination with the use of user-generated content. Moreover, lemmatization was also shown to have beneficial effects on accuracy [65].

Several models built in the field of sentiment analysis, both modern [66] and older [67,68], have shown to rely on the most disparate combination of pre-processing techniques, such as negation and punctuation [69] or pos tagging [70].

Furthermore, Pradha et al. [71] proposed an effective technique for pre-processing text data and developed an algorithm to train Support Vector Machine (SVM), Deep Learning (DL) and Naïve Bayes (NB) classifiers for processing Twitter data, developing an algorithm to weight the feeling evaluation in relation to the weight of the hashtag and clean text. Sohrabi and Hemmatian [72] presented an efficient pre-processing method for opinion mining, testing it on Twitter user comments, and demonstrated how its use in combination with SVM and ANNs achieves the highest accuracy scores compared to other methods. Alam and Yao [73] studied the impact of pre-processing on the accuracy of three machine learning algorithms for sentiment analysis, i.e., NB, SVM and Maximum Entropy (MaxE), demonstrating that in the case of the NB algorithm, accuracy is significantly improved after the application of the pre-processing phases of the text.

Moreover, Babanejad et al. [74] made a complete analysis of the role of pre-processing techniques but, for the first time, not in affective systems but in models based on word vectors applied to affective systems, giving significant insights on each pre-processing technique when implemented in the training phase and/or in the downstream task phase.

The techniques described above were more recently applied to language models for the sentiment analysis of tweets. In particular, Azzouza et al. [11] proposed TwitterBERT, a four-phase framework for twitter sentiment analysis, including the unicode code of emoticons and emojis during the pre-training phase avoiding a specific pre-processing phase. This work, by contrast, proposes to exploit both pre-trained language models on plain text and to pre-process emoticons and emojis by transforming them into text rather than integrating their unicode encoding.

### 2.3. Sentiment Analysis in the Italian Language

Regarding the Italian scene, the number of annotated corpora is much lower, although recent work on the classification of texts focused on approaches based on deep learning [75], possibly starting from models pre-trained on large unlabeled resources. With regard to the scope of analysis of tweets collected by the social network Twitter, it is possible to identify several problems due to differences in structure and grammar compared to plain text. In fact, there are several scientific works related to this issue, e.g., Vassallo et al. [76]. For example Deriu and Cieliebak [77] proposed a solution based on a 2-layer CNN, using 9-fold cross validation and combining the outputs of the nine resulting classifiers to increase robustness, and obtained the best score (with an amended run) in *SENTIPOLC Task 2: Polarity* classification at the *Evaluation of NLP and Speech Tools for Italian 2016* (EVALITA 2016) [17].

The three best performing systems participating in the EVALITA 2016 SENTIPOLC *Task 2: Polarity Classification* were used as baselines for comparison. In particular, these are those of the UniPI, Unitor and ItaliaNLP teams headed respectively by the University of Pisa, the University of Rome Tor Vergata and the ItaliaNLP Lab of the Institute for Computational Linguistics (ILC), part of the National Research Council of Italy (CNR). The UniPI team adopted a deep learning method that required the modeling of individual tweets through both WE and CNN (system named *UniPI.2.c* by Attardi et al. [78]). The Unitor team took a similar approach to the UniPI team, using an extended representation of tweets with additional features taken from the Distributional Polarity Lexicons in combination with a CNN (systems named *Unitor.1.u* and *Unitor.2.u* by Castellucci et al. [79]). The ItaliaNLP team used a SVM learning algorithm paired to an LSTM network based on specific linguistic and semantic feature engineering and existing external resources, such as lexicons specific for sentiment analysis tasks (system named *ItaliaNLP.1.c* by Cimino and Dell’Orletta [80]).

Further analyses were made on SENTIPOLC 2016 Task 2 after this challenge: Mattei et al. [81] presented a mixed single- and multi-task learning approach that is able to improve the performance in polarity detection by up to a 0.698$F_{1}$ score. Magnini et al. [82] used multilingual BERT, obtaining results that are not competitive with the state-of-the-art: according to Pires et al. [83], multilingual BERT is able to perform multilingual adaptation but deeper fine-tuning is needed when the task is more related to semantics. Instead, Petrolito and Dell’Orletta [84] investigated in detail the best way to manage non-BERT word embedding.

Finally, one of the the most interesting results is certainly AlBERTo, a model based on BERT but specifically trained on a large unlabeled tweet corpus [13]: after performing its fine-tuning on EVALITA 2016 tasks, it reached state-of-the-art performances. However, although the effectiveness of BERT-based models has been widely demonstrated, it has not yet been experimentally demonstrated that pre-training on tweet corpora is the best approach to handle scenarios that have a grammar and syntax based on specific elements. In the following an alternative approach is proposed with tweets in the Italian language, which focuses on the pre-processing phase in order to make the most of a model based on BERT but trained on generic corpora.

## 3. Methods

**Figure 1:**
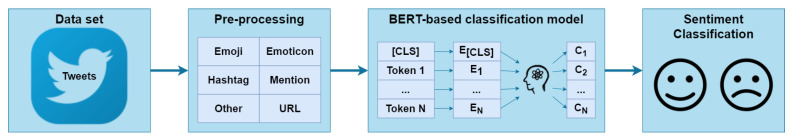
Pipeline overview. *[CLS]* is the BERT special classification token, *E* is short for *Embedding*, and *C* and *T* are the final hidden states given by the transformers architecture.

In this section the proposed approach to classifying tweets and making the sentiment analysis is illustrated. It is essentially a two-step pipeline as shown in Figure 1. In detail, the first step of the pipeline consists in applying a set of pre-processing procedures to convert the Twitter jargon into plain text, including emojis and emoticons, while the second step places the data thus processed into the classification system based on the BERT language model that has been pre-trained on plain text corpora. In particular, the proposed pre-processing procedures are outlined in Section 3.1, the architecture of the classification model adopted is explained in Section 3.2 and the fine-tuning modalities of this system are described in Section 3.3.

### 3.1. Pre-Processing Procedures

The raw tweets collected on Twitter using its API (https://developer.twitter.com/en/docs/api-reference-index) generally result in a very noisy and obscure dataset, due to people’s random and creative use of social media [85]. Tweets have certain special features, i.e., emojis, emoticons, hashtags and user mentions, coupled with typical web constructs, such as email addresses and URLs, and other noisy sources, such as phone numbers, percentages, money amounts, time, date, and generic numbers. In this article a set of pre-processing procedures, which has been tailored to *translate* tweets into sentences constructed in a more conventional form, is adopted. The sequence of actions of each pre-processing procedure to transform all the mentioned noisy sources is described below. These procedures are described here for the case study of the Italian language, but they are either language-independent or based on linguistic resources, i.e., conversion tables, also existing for other languages.

Firstly, most of the noisy entities are normalized because their particular instances generally do not contribute to the identification of the feeling within a sentence. Regarding *date*, *email addresses*, *money amounts*, *numbers*, *percentages*, *phone numbers* and *time*, this process is performed by using the ekphrasis tool (https://github.com/cbaziotis/ekphrasis) [47], which enables to individuate regular expressions and replace them with normalized forms, through the following rules:

^([0-2][0-9]|(3)[0-1])(\/)(((0)[0-9])|((1)[0-2]))(\/)\d{4}$→*<date>*

It validates the date format *dd/mm/yyyy*, where *dd* ranges from 01 to 31, *mm* ranges from 01 to 12 and *yyyy* can be any sequence of four digits.

(\w+@\w+.[\w+]{2,4}$)→*<email>*

It validates the email format *a@b.c*, where the lengths *a* and *b* are arbitrary and *c* must be between 2 and 4 characters long.

(^\d∗(\.\d{1,2})?$)→*<money>*

It validates the money format, which could be an integer, an integer with 1 or 2 decimal places or just something like *.xy* where x and y are decimal places.

^[0-9]∗$→*<number>*

It validates a contiguous string of digits.

\d+(\%|\s\bpercent\b)→*<percentage>*

It validates the percentage format that could appear as *x%* or *x percent*, where *x* is an arbitrary integer.

([(][\d]{3}[)][ ]?[\d]{3}-[\d]{4})→*<phone>*

It validates the phone format *(DDD) DDD-DDDD*, where *D* is any digit.

^([0-1][0-9]|[2][0-3]):([0-5][0-9])$→*<time>*

It validates the time format *HH:MM*, where *HH* ranges from 00 to 23, while *MM* ranges from 00 to 59.

Similarly, Uniform Resource Locators (URLs), often used in tweets to share hypertext links to other web pages, do not contribute to the classification of a text, but can lead to an incorrect classification if they contain conflicting words: therefore all URLs in the tweets are normalized. For this purpose, a function is used to search for all URLs corresponding to the following regular expression and replace them with the word *url*:

(\w+:\/\/\S+) → *url*

It validates the URL format *a://b* where *a* is any set of characters and *b* is any non-whitespace set of characters.

Each Twitter user is associated with a unique username. Users often *mention* other users in their tweets by using a *@* followed by the unique username. All user mentions are replaced in this case with the *@user* token. This normalization is applied by identifying and replacing user mentions through a regular expression:

(@[A-Za-z0-9]+) → *@user*

It validates the mention format *@Aa1*, where *Aa1* indicates any alphanumeric set of characters.

*Hashtags* are phrases preceded by the hash symbol *#* and without spaces. The proposed procedure, coherently with previous approaches, consists in applying a tokenization. The python wordninja library (https://github.com/keredson/wordninja) is used due to its support both for loading custom dictionaries for word definition and for multilingual applications. Specifically, the employed Italian dictionary is available online (https://dizionari.repubblica.it/italiano.html) and counts more than 500,000 entries. Moreover, a custom module has been developed to make it compatible with the library mentioned above. The resulting phrase is placed between two special characters, *<* and *>*, to keep it grouped within the tweet but separate from the rest:

(#S+) → *< tokenize(S+) >*

It validates the hashtag format *#aa*, where *#* matches the corresponding character, while *aa* matches any non-whitespace set of characters.

*Emoticons* are short sequences of symbols, letters or numbers intended to represent certain facial expressions and postures. Emoticons are used within social media to communicate moods: therefore, in most cases, they are strongly bound to the overall sentiment. Differently from other works, in this paper, these elements are proposed to be translated into a word that expresses the same mood. The complete list of the emoticons considered, which are the most recurrent, is shown in Table 1.

*Emojis*, introduced in 1997, are elements of a standardized set of small pictorial glyphs depicting different items, from smiling faces to international flags, and have seen a drastic increase in usage in social media over the last decade [86]. Among them, some emojis represent universal sentiment expressions [87]. Similarly to what was just described for emoticons and differently from other works, emojis are proposed to be transformed so that they remain consistent within the sentence. In order to do this, the international table of officially recognized emojis in Italian (https://emojiterra.com/it/punti-di-codice/) is used, and a custom python module has been developed for searching for and substituting emojis according to the mentioned international table. Some examples are reported in Table 2.

Although the text conversion tables for emoticons (Table 1) and emojis (Table 2) used are specific for the Italian language, they also exist for other languages.

Finally, this approach keeps *punctuation* and leaves capital letters untouched, not lower-casing them. These choices consider the BERT architecture used and its pre-training on raw text that comprises punctuation and upper-cased characters. Hereafter an example of complete pre-processing, where:

*#serviziopubblico: La ’buona scuola’ dev’essere: fondata sul lavoro…allora i politici tutti ripetenti? Si, Mastella prima di tutti 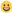
http://a.co/344555*

(*#publicservice: The ’good school’ must be: based on work…then politicians all repeating? Yes, Mastella above all 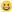
http://a.co/344555*)

is transformed into the following:

*<servizio pubblico>: La ’buona scuola’ dev’essere: fondata sul lavoro…allora i politici tutti ripetenti? Si, Mastella prima di tutti Faccina Con Un Gran Sorriso url*

(*<public service>: The ’good school’ must be: based on work…then politicians all repeating? Yes, Mastella above all Grinning Face url*).

### 3.2. Bert System Architecture

**Figure 2:**
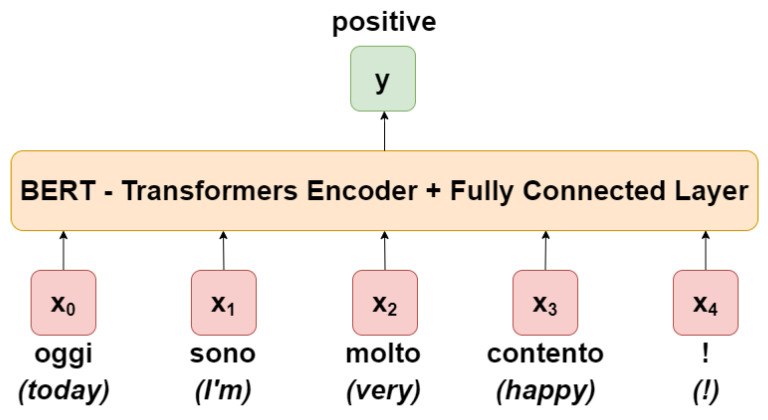
BERT architecture overview.

Among modern language modeling architectures, BERT [9] is one of the most popular. Its generalization capability is such that it can be adapted to different down-stream tasks according to different needs, be it NER or relation extraction, question answering or sentiment analysis. The core of the architecture is trained on particularly large text corpora and, consequently, the parameters of the most internal layers of the architecture are frozen. The outermost layers are instead those that adapt to the task and on which the so-called fine-tuning is performed. A simplified overview is shown in Figure 2.

Going into detail, one can distinguish two main architectures of BERT, the base and the large. The architectures differ mainly in four fundamental aspects: the number of hidden layers in the transformer encoder, also known as transformer blocks (12 vs. 24), the number of attention heads, also known as self-attention [38] (12 vs. 16), the hidden size of the feed-forward networks (768 vs. 1024) and finally the maximum sequence length parameter (512 vs. 1024), i.e., the maximum accepted input vector size. In this article the base architecture is used, and the corresponding hyper-parameters are reported in Table 3.

In addition, the BERT architecture employs two special tokens: *[SEP]* for segment separation and *[CLS]* for classification, used as the first input token for any classifier, representing the whole sequence and from which an output vector of the same size as the hidden size *H* is derived. Hence, the output of the transformers, i.e., the final hidden state of this first token used as input, can be denoted as a vector $C\in R^{H}$.

The vector C is used as input of the final fully-connected classification layer. Given the parameter matrix $W\in R^{KxH}$ of the classification layer, where *K* is the number of categories, the probability of each category *P* can be calculated by the softmax function as:

(1) $$P=softmax(CW^{T}).$$

#### Transformer

The transformer [38] is the base of BERT. Consider **x** and **y** as a sequence of sub-words taken from two sentences. The [CLS] token is located before **x**, while the [SEP] token is located after both **x** and **y**. Say *E* is the embedding function and *LN* is the normalization layer [38], the embedding is obtained through:

(2) $${\hat{h}}_{i}^{0}=E\left(x_{i}\right)+E\left(i\right)+E\left(1_{x}\right)$$

(3) $${\hat{h}}_{j+|x|}^{0}=E\left(y_{j}\right)+E\left(j+|x|\right)+E\left(1_{y}\right)$$

(4) $${\hat{h}}_{.}^{0}=Dropout\left(LN\left({\hat{h}}_{.}^{0}\right)\right)$$

Then the embeddings are passed through *M* transformer blocks. Using the Feed Forward (FF) layer, the element-wise Gaussian Error Linear Units (GELU) activation function [88] and the Multi-Heads Self-Attention (MHSA) function, in each transformer block it is valid that:

(5) $${\hat{h.}}^{i+1}=Skip\left(FF,Skip\left(MHSA,h.^{i}\right)\right)$$

(6) Skip(f,h)=LN(h+Dropout(f(h)))

(7) $$FF\left(h\right)=GELU\left(hW_{1}^{⊤}+b_{1}\right){W_{2}}^{⊤}+b_{2}$$

where $h^{i}$∈R^(|**x**|+|**y**|)×d_h_^, $W_{1}$∈R^4dh×d_h_^, $b_{1}$∈R^4d_h_^, $W_{2}$∈R^4dh×d_h_^, $b_{2}$∈R^4d_h_^ and each new ${\hat{h}}_{i}$ position is equal to:

(8) $$\begin{array}{c} \left[\ldots ,{\hat{h}}_{i},\ldots \right]=MHSA\left(\left[h_{1},\ldots ,h_{\left|x|+|y\right|}\right]\right)=\\ W_{o}Concat\left(h_{i}^{1},\ldots ,h_{i}^{N}\right)+b_{o} \end{array}$$

Instead, in each attention head it is valid that:

(9) $$h_{i}^{j}=\sum_{k=1}^{\left|x|+|y\right|}Dropout\left(\alpha_{k}^{\left(i,j\right)}\right)W_{V}^{j}h_{k}$$

(10) $$a_{k}^{\left(i,j\right)}=\frac{exp\frac{{\left(W_{Q}^{j}h_{i}\right)}^{⊤}W_{K}^{j}h_{k}}{\sqrt{d_{h}/N}}}{\sum_{k^{\prime }=1}^{\left|x|+|y\right|}exp\frac{{\left(W_{Q}^{j}h_{i}\right)}^{⊤}W_{K}^{j}h_{k^{\prime }}}{\sqrt{d_{h}/N}}}$$

where $h_{i}^{j}$∈$R^{\left(d_{h}/N\right)}$, $W_{o}$∈R^dh×d_h_^, $b_{o}$∈R^d_h_^ and $W_{Q}^{j}$,$W_{K}^{j}$,$W_{V}^{j}$∈R^d_h_/N×d_h_^, with *N* equal to the number of attention heads.

### 3.3. Model Training

The whole classification model has been trained in two steps, involving firstly the pre-training of the BERT language model and then the fine-tuning of the outermost classification layer.

The Italian BERT model used with the Hugging Face framework is made available by the MDZ Digital Library team at the Bavarian State Library (https://huggingface.co/dbmdz/).

The Italian BERT XXL is pre-trained on two corpora: on the one side the source data consist of a recent Wikipedia dump and various texts from the OPUS corpora (http://opus.nlpl.eu/) collection with a final corpus size equal to about 13 GB and more than 2 billions tokens, while on the other side the source data are further extended with data from the Italian part of the OSCAR corpus (https://traces1.inria.fr/oscar/) with a final corpus size equal to about 81 GB and more than 13 billions tokens. The cased version was chosen, being more suitable for the proposed pre-processing method.

The fine-tuning of the model was performed by using labelled tweets comprising the training set of the employed dataset. In particular, the fully connected classification layer was learned accordingly. During training the loss function used was categorical cross-entropy. For this study, the hyper-parameters used are shown in Table 3. The maximum sequence length was reduced to 128 due to the short length of the tweets.

## 4. Experimental Design

In this section, the dataset employed here to train and test the described classification model is illustrated in Section 4.1, while the evaluation metrics adopted to assess its performance are reported in Section 4.2. Finally, Section 4.3 describes the experimental setup and execution.

### 4.1. Data Set

In order to have a comparison with the state-of-the-art on sentiment analysis, the proposed approach was tested on the most famous Italian dataset that exists in the literature, the SENTIPOLC 2016, also having the possibility of comparison with the state-of-the-art and in particular with AlBERTo [13], which is essentially an Italian BERT model trained from scratch directly on a corpora of tweets. In detail, this dataset was built from the following corpora:

1. TW-SENTIPOLC14 [89];
2. TWitterBuonaScuola [90];
3. Tweets selected from the TWITA 2015 collection [91];
4. Tweets collected in the context of the EVALITA 2016 NEEL-IT Task [92].

In particular, starting from the listed datasets, SENTIPOLC 2016 was reorganized in order to be a uniform corpus of tweets with respect to the new annotation guidelines specifically prepared for the tasks scheduled during the Evalita 2016 SENTIPOLC Task [17], which were:

- Task 1: Subjectivity Classification. It was intended to verify the subjectivity and objectivity of tweets.
- Task 2: Polarity Classification. Its purpose was to verify positivity, negativity and neutrality (and their mixes) in tweets. This paper focuses on this task.
- Task 3: Irony Detection. It aimed to verify whether tweets are ironic or not.

In Table 4, the main aspects regarding the composition of the dataset, i.e., the presence of emojis, emoticons, hashtags, mentions and urls in tweets, are shown.

Within the adopted annotation scheme there are six tags: *iro* (ironic), *lneg* (literally negative), *lpos* (literally positive), *oneg* (overall negative), *opos* (overall positive) and *subj* (subjective) but, for the purposes of this article, only *oneg* and *opos* tags are considered. These tags assume a value 0 or 1 respectively in the absence or presence of the relative sentiment.

In this work, overall sentiment is analyzed; therefore, two classification tasks are considered, one detecting the presence of positive sentiment, where the output *opos* could be class 1 if positive sentiment is detected, or class 0 otherwise; in the other task, the output *oneg* could be class 1 if negative sentiment is detected, or class 0 otherwise. As a consequence, for *opos* class 1 means positive or mixed sentiment, and class 0 means neutral or negative sentiment, while for *oneg* class 1 means negative or mixed sentiment, and class 0 means neutral or positive sentiment. Table 5 reports the labels’ distribution.

### 4.2. Metrics

According to the official evaluation system presented at EVALITA 2016 [17], positive and negative polarities are evaluated here independently as two separate classification tasks and, for each task, the precision *P* (11), the recall *R* (12) and the $F_{1}$ score (13) are computed. Since it is necessary to indicate the $F_{1}$ score both for class 0 and class 1 and in order to avoid an unclear notation, the 0 and 1 subscripts will be used for this purpose, assuming that the $F_{1}$ score will continue to be used, both in the case of $F_{0}$ notation (not to be confused with $F_{0}$ score) and in the case of $F_{1}$ notation. In detail:

(11) $$P_{c}^{p}=\frac{correct_{c}^{p}}{assigned_{c}^{p}}$$

(12) $$R_{c}^{p}=\frac{correct_{c}^{p}}{total_{c}^{p}}$$

(13) $$F_{c}^{p}=2\frac{P_{c}^{p}R_{c}^{p}}{P_{c}^{p}+R_{c}^{p}}$$

where *p* indicates the polarity, both positive (it will be indicated as *pos*) and negative (it will be indicated as *neg*), while *c* stands for the considered class (0 or 1). Moreover, the $F_{1}$ score (14) for each task is computed as the average of the $F_{1}$ scores of the respective pair of classes:

(14) $$F^{p}=\frac{F_{0}^{p}+F_{1}^{p}}{2}$$

Finally, the overall $F_{1}$ score (15) is given by the average of the $F_{1}$ scores relative to the two polarities:

(15) $$F=\frac{F^{pos}+F^{neg}}{2}$$

### 4.3. Experiments Execution

In order to evaluate the effectiveness of the pre-processing step within the whole pipeline, different experiments were conducted by including this step or not. In particular, three experiments were performed with each configuration, to average fluctuations due to intrinsic randomness of results.

Each experiment was performed as follows. The training part of the dataset described in Section 4.1 was first pre-processed and then used to feed the model fine-tuning, as described in Section 3.3. Two models were fine-tuned for each experiment, i.e., the models to detect positive and negative sentiments. Fine-tuning was executed on an IBM POWER9 cluster with NVIDIA V100 GPUs. Then, each tweet of the test set of the employed dataset was eventually pre-processed and fed to the model, and the official evaluation system (https://github.com/evalita2016/data/blob/master/sentipolc/sentipolc16_evaluation.zip) released with the SENTIPOLC 2016 dataset was used to compute the metrics described in Section 4.2. The values obtained for each of the metrics by repeated experiments with the same configuration were averaged, and the mean values are reported as results.

## 5. Results and Discussion

In order to compare the results of the proposed approach with the state-of-the-art systems described in Section 2.3, a summary of the scores is reported in Table 6. $F_{1}$ scores obtained by the different systems for the classification of both positive and negative sentiments are listed, as well as the combined $F_{1}$ score.

In addition to AlBERTo and to the participants in the challenge, we have also included the results of further systems: the CNN-based system by Deriu and Cieliebak [77], the LSTM-based system by Mattei et al. [81] and the multilingual BERT-based system by Magnini et al. [82].

The proposed approach allows to obtain an improvement compared to the best system, i.e., AlBERTo, in the order of almost 3% on average, showing the effectiveness of the procedure without a pre-training directly on tweets. The reason is to be found in two main and closely interrelated issues: (1) the use of a version of BERT that has enjoyed an extremely large pre-training corpus, due to the choice to use plain text instead of a collection of tweets, and (2) the use of a set of pre-processing procedures that have added, to classical transformations performed on the source text, the conversion of emojis and emoticons: consequently, all of the conveyed sentiment has been transferred into plain text, i.e., the best representation for being processed by the pre-trained version of BERT used here.

In confirmation of these results, an ablation study was performed, first disabling the pre-processing step, thus using only the BERT classification model, and successively enabling both the pre-processing step and the BERT classification model.

The results of this ablation study are detailed in Table 7, for positive and negative polarity, with values of precision *P*, recall *R* and $F_{1}$ score for both classes 0 and 1.

In detail, it is possible to note that the usage of both the pre-processing procedures and the BERT classification model allows to obtain, on the one hand, better precision and lower recall for class 0 and, on the other hand, lower precision and better recall for class 1, related to the positive case. This suggests that the proposed pre-processing influences the classification of positive sentiment by increasing the number of tweets in which positive characteristics are detected. Instead, for the negative case, results obtained are appreciably better than using only the BERT classification model. In particular, a better precision and a comparable recall for class 0 and a comparable precision and a better recall for class 1 can be achieved. This means that the proposed pre-processing procedures influence the classification of negative sentiment by correctly identifying the presence of negative characteristics, which are not identified by using only the BERT classification model.

Summarizing, the combined $F_{1}$ score of only BERT is equal to F=0.7437, and the average $F_{1}$ obtained in the three experiments with pre-processed tweets is F=0.7500. Furthermore, the standard deviation among the three experiments below each reported media is equal to 0.0018. A two-tailed t-test with α=0.05 revealed that the result using only BERT was significantly improved by using the proposed pre-processing.

In the following, some examples of tweets that were incorrectly classified by using only the BERT classification model and correctly classified by the model that also uses the proposed pre-processing procedures are reported: the latter allows a better structuring of the tweets that is closer to the plain text and therefore is better managed by the the BERT classification model. In detail, considering the following tweet:

*Alla ’Buona scuola’ noi rispondiamo con la ’Vera scuola’! #noallabuonascuola #laverascuola*

(*To the ’Good school’ we respond with the ’True school’! #noallabuonascuola #laverascuola*)

It is possible to see how, without any pre-processing, the hashtags are classified as unknown words, giving no contribution to the classification; the model is fooled by the word “Buona” (“Good”) and classifies the tweet with class 1 for positive sentiment and 0 for negative sentiment. Conversely, after pre-processing, the tweet becomes:

*Alla ’Buona scuola’ noi rispondiamo con la ’Vera scuola’! <no alla buona scuola> <la vera scuola>*

(*To the ’Good school’ we respond with the ’True school’! <no to good school> <the real school>*)

In the hashtag, the presence of the word “no” near the word “buona” (“good”) inverts the sense of the latter word, influencing the classification: this time the model classifies the tweet with class 0 for positive sentiment and 1 for negative sentiment. Another example is the following:

*#AndreaColletti #M5S: #Riforma della #prescrizione https://t.co/iRMQ3x5rwf #Incalza #TuttiInGalera #ersistema #terradeifuochi*

(*#AndreaColletti #M5S: #Reformation of the #prescription https://t.co/iRMQ3x5rwf #Pressing #AllInJail #thesystem #fireland*)

Classified as neutral (0 for both polarities) by BERT, since it is entirely composed of hashtags not encountered during training (except for the “#M5S” that however does not give any contribution of polarity). The pre-processing transforms the tweet as follows:

*<Andrea Colletti> <M5S>: <Riforma> della <prescrizione> url <Incalza> <Tutti In Galera> <er sistema> <terra dei fuochi>*

(*<Andrea Colletti> <M5S>: <Reformation> of the <prescription> url <Pressing> <All In Jail> <the system> <fire land>*)

Where the phrases “Tutti In Galera” (“All in Jail”) and “terra dei fuochi” (“fire land”) are correctly understood: in fact the model correctly identifies the negative sentiment of the tweet and classifies it as class 1 for negative polarity. Another example highlights the advantages of translating emojis:

*#Roma #PiazzaDiSpagna pochi minuti fa 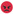. #NoComment #RomaFeyenoord http://t.co/2F1YtLNc8z*

(*#Roma #PiazzaDiSpagna few minutes ago 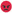. #NoComment #RomaFeyenoord http://t.co/2F1YtLNc8z*)

Is classified as neutral (0 for both polarities) by only BERT, since the emoji is an unknown word. By applying the pre-processing step, the tweet is changed to:

*<Roma> <Piazza Di Spagna> pochi minuti fa Faccina Arrabbiata. <No Comment> <Roma Feyenoord> url*

(*<Rome> <Piazza Di Spagna> a few minutes ago Angry Face. <No Comment> <Roma Feyenoord> url*)

Where the insertion of the text “Faccina Arrabbiata” (“Pouting Face”) in place of the emoji expresses the negative feeling associated with it and, as a consequence, the model classifies the tweet as class 1 for negative polarity.

## 6. Conclusions

The objective of this work was the introduction of an effective approach based on the BERT language model for Twitter sentiment analysis. It was arranged in the form of a two-step pipeline, where the first step involved a series of pre-processing procedures to transform Twitter jargon, including emojis and emoticons, into plain text, and the second step exploited a version of BERT, which was pre-trained on plain text, to fine-tune and classify the tweets with respect to their polarity. The use of language models pre-trained on plain texts rather than on tweets was motivated by the necessity to address two critical issues shown by the scientific literature, namely (1) pre-trained language models are widely available in many languages, avoiding the time-consuming and resource-intensive model training directly on tweets from scratch, allowing to focus only on their fine-tuning; (2) available plain text corpora are larger than tweet-only ones, allowing for better performance. A case study describing the application of this approach to the Italian language was presented. The results revealed notable improvements in sentiment classification performance, both with respect to other state-of-the-art systems and with respect to the usage of only the BERT classification model. Even though the approach was assessed for the Italian language, its strength relies on its generalizability to other languages other than Italian, thanks to pre-processing procedures that are language-independent or easily re-applicable to other languages, and the usage of pre-trained language models, which exist for many languages and, thus, do not require the time-consuming and resource-intensive model retraining directly on big corpora of tweets to be performed. Given these considerations, the approach has a general basis from a methodological perspective and can be proficiently applied also to other languages.

Future work will be directed to investigate the specific contributions of each pre-processing procedure, as well as other settings associated with the tuning, so as to further characterize the language model for the purposes of sentiment classification. Moreover, possible future extensions of this work include the application of the proposed approach for similar sentiment-related tasks like irony detection and subjectivity classification, in order to validate its effectiveness with particular focus on the pre-processing step. Finally, the proposed approach will also be tested and assessed with respect to other datasets, languages and social media sources, such as Facebook posts, in order to further estimate its applicability and generalizability.

## Figures and Tables

**Table 1:** Transformation of emoticons.

Symbol	English	Italian
:) :-) 8-) :-] :-))	Happy	Felice
:-( :( :-\	Sad	Triste
:-P x-p	Joking	Scherzo
<3 < 3 :∗	Love	Amore

**Table 2:** Examples of emoji transformation.

Emoji	Meaning	Italian
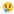	Crying Face	Faccina Che Piange
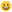	Grinning Face	Faccina Con Un Gran Sorriso
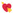	Heart With Arrow	Cuore Con Freccia
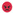	Pouting Face	Faccina Arrabbiata

**Table 3:** Hyper-parameters of the fine-tuned Italian BERT XXL Cased.

Hyperparameter	Value
Attention heads	12
Batch size	8
Epochs	5
Gradient accumulation steps	16
Hidden size	768
Hidden layers	12
Learning rate	0.00003
Maximum sequence length	128
Parameters	110 M

**Table 4:** SENTIPOLC 2016 dataset composition.

Characteristic	Train	Test
Emoji	157	145
Emoticon	320	20
Hashtag	5417	2180
Mention	3138	1564
Other	1468	464
URL	2314	956

**Table 5:** Label distribution.

Combination		Resulting Sentiment	Train	Test
oneg	opos						
0	0	Neutral	2816	914
0	1	Positive	1611	316
1	0	Negative	2543	734
1	1	Mixed	440	36

**Table 6:** State-of-the-art comparison.

System	$F^{\mathrm{pos}}$	$F^{\mathrm{neg}}$	*F*
Proposed System	**0.7381**	**0.7620**	**0.7500**
AlBERTo	0.7155	0.7291	0.7223
LSTM-based [81]	0.6600	0.7360	0.6980
CNN-based [77]	0.6529	0.7128	0.6828
UniPI.2.c	0.6850	0.6426	0.6638
Unitor.1.u	0.6354	0.6885	0.6620
Unitor.2.u	0.6312	0.6838	0.6575
ItaliaNLP.1.c	0.6265	0.6743	0.6504
Multilingual BERT [82]	-	-	0.5217

**Table 7:** Classification results.

Model	$P_{0}^{\mathrm{pos}}$	$R_{0}^{\mathrm{pos}}$	$F_{0}^{\mathrm{pos}}$	$P_{1}^{\mathrm{pos}}$	$R_{1}^{\mathrm{pos}}$	$F_{1}^{\mathrm{pos}}$	$F^{\mathrm{pos}}$
BERT	0.9172	0.8871	0.9019	0.5419	0.6250	0.5805	0.7412
Pre-processing + BERT	0.9262	0.8618	0.8928	0.5125	0.6780	0.5833	0.7381
**Model**	**$P_{0}^{\mathrm{neg}}$**	**$R_{0}^{\mathrm{neg}}$**	**$F_{0}^{\mathrm{neg}}$**	**$P_{1}^{\mathrm{neg}}$**	**$R_{1}^{\mathrm{neg}}$**	**$F_{1}^{\mathrm{neg}}$**	**$F^{\mathrm{neg}}$**
BERT	0.7639	0.9285	0.8382	0.8257	0.5416	0.6541	0.7461
Pre-processing + BERT	0.7759	0.9295	0.8458	0.8358	0.5710	0.6782	0.7620

## Data Availability

The data presented in this study are openly available at http://www.di.unito.it/~tutreeb/sentipolc-evalita16/data.html.
